# The College of Physicians in Vienna and its Jewish members after Austria’s annexation (*Anschluss*) to Nazi Germany in 1938

**DOI:** 10.1007/s00508-026-02723-x

**Published:** 2026-03-10

**Authors:** Josef Hlade, Beatrix Volc-Platzer, Hermann Zeitlhofer, Teresa Lang, Herwig Czech

**Affiliations:** 1 Institut für Ethik, Sammlungen und Geschichte der Medizin, Medizinische Universität Wien, Währinger Straße 25, 1090 Vienna, Austria; 2 College of Physicians in Vienna (President 2020–2024), Frankgasse 8, 1090 Wien, Österreich; 3 College of Physicians in Vienna, Frankgasse 8, 1090 Wien, Österreich; 4 College of Physicians in Vienna, Frankgasse 8, 1090 Wien, Österreich; 5 Institut für Ethik, Sammlungen und Geschichte der Medizin, Medizinische Universität Wien, Währinger Straße 25, 1090 Wien, Österreich

**Keywords:** Vienna Medical School, National Socialism, Holocaust, Antisemitism, Jewish physicians

## Abstract

This text outlines the process by which Jewish members of the College of Physicians in Vienna (*Gesellschaft der Ärzte in Wien*) were disenfranchised and excluded subsequent to Austria’s annexation to the German Reich (euphemistically called the *Anschluss*) in March 1938. At the beginning of 1938, 60% of the members and about one third of the board were of Jewish origin. The provisional director, Adolf Irtl (1867–1947), along with members of the Medical Faculty at the University of Vienna and the Nazi Party, discussed how to deal with and expel the Jewish members. Their membership rights were restricted from the outset. Following the *Anschluss*, the College of Physicians retroactively demanded “unpaid membership fees” from Jewish members, threatening their already precarious financial situation. In February 1939, the Medical Society of Vienna (*Wiener Medizinische Gesellschaft*) was founded as a Nazi replacement organization. Jewish members were no longer accepted. As part of the project “History of the College of Physicians in Vienna: the critical years 1930–1960”, the names of all those who were expelled for antisemitic or political reasons have been documented. Some of these victims are presented here as examples.

## History of the College of Physicians in Vienna: the critical years 1930–1960

Following Austria’s *Anschluss* to the German Reich on 13 March 1938, Jewish doctors were promptly forced to leave their profession and ultimately to flee Austria. Those who could not escape were persecuted and murdered by the Nazis unless they found a way to survive. In Vienna, 3400 out of 4900 doctors lost their licence to practise and 40% of lecturers and professors at the University of Vienna were persecuted. The Medical Faculty was hit particularly hard, losing around 52% of its teaching staff. No other Medical Faculty in the German-speaking world suffered a greater brain drain [1, 2]; however, discrimination had begun many years earlier. Antisemitism was firmly established at the University of Vienna by the time of the First World War. As early as the 1920s, networks of antisemitic professors were preventing the academic careers of Jewish and left-leaning scientists. Academic merit became less important than “Aryan descent” and right-wing convictions [3–13]. These antisemitic networks had also prevailed at the Medical Faculty since at least the mid-1920s, making it virtually impossible for Jewish physicians to attain a full professorship. Unlike at other faculties, Jewish physicians were still able to obtain habilitations in certain fields at the Medical Faculty until the late 1920s and early 1930s [3] but in many fields, such as surgery, even an associate professorship had become an unrealistic goal.^1^

Founded in 1837, the College of Physicians in Vienna is one of the oldest medical societies in the world, after the Royal College of Physicians (founded 1518), the Medical Society of London (founded 1773) and the College of Physicians of Philadelphia (founded 1787). Some of the most prominent figures in Viennese medicine have led it, including the pathologist Carl von Rokitansky (1804–1878), the surgeon Theodor Billroth (1829–1894), the physiologist Siegmund Exner (1846–1926) and the surgeon Anton von Eiselsberg (1860–1939) [14].

The College was founded in the Metternich era. It was the central place in Vienna for discussions and debates in the medical sciences. During the Metternich era, the Faculty of Medicine was very restricted in this respect, so a forum for scientific exchange was needed. The College retained this function in subsequent eras. This explains its unique and outstanding position in Viennese medicine. Even before Rokitansky’s presidency (1850), the College was the mouthpiece of the Second Vienna Medical School (or Younger Vienna Medical School), which had a major influence on the development of the scientific worldview in medicine [15, 16]. One of the College’s early highlights was its commitment in 1847 to disseminating the groundbreaking discovery of Ignaz Semmelweis (1818–1865) on the prevention of childbed fever. The College was the first to present these findings to the world. The exponents of the Second Vienna Medical School, who already held key positions within the College, e.g., Josef Škoda (1805–1881) and Ferdinand Hebra (1816–1880), proved to be innovators as they publicly supported Semmelweis despite great mistrust within the Viennese Faculty and controversy surrounding his findings at the time [17, 18]. From then on, the College functioned as an important forum for Viennese medicine. In 1893, the College moved into its own building. It was renamed Billrothhaus in 1919, in honour of Theodor Billroth, who initiated its creation [15].

Researchers endeavored to be accepted by the College’s board to present original medical papers at meetings, with the subsequent aim of publishing them in the College’s journal, the *Wiener klinische Wochenschrift* (founded in 1888), which was of outstanding importance to the scientific community at the time. Since then, numerous groundbreaking findings related to Viennese medicine have either been presented for the first time in the College of Physicians or published in the *Wiener klinische Wochenschrift*. The College of Physicians was also an important meeting place for researchers because it had the most important library of medical literature in Vienna. In 1938, the library contained more than 22,000 monographs and around 1300 different journals [19, 20].

The ear, nose and throat (ENT) specialist Robert Bárány (1914), the neurologist and psychiatrist Julius Wagner-Jauregg (1927) and the pathologist Karl Landsteiner (1930), Nobel Prize winners for physiology or medicine who trained in Vienna, were closely associated with the College of Physicians in Vienna. The lectures held at the College and their subsequent publication in the *Wiener klinische Wochenschrift* were, in some cases, of major importance to their careers. For example, Karl Landsteiner’s discovery of blood groups was published in the *Wiener klinische Wochenschrift* in 1901, following various lectures at the College [21, 22]. Bárány, for example, demonstrated his Bárány’s pointing test (*Zeigeversuch*) at the College of Physicians on 10 June 1910, just a few days after his first presentation at a neurological conference in Baden-Baden [15, 24]. One of his first papers on the physiology and pathology of the vestibular apparatus was published in the *Wiener klinische Wochenschrift* [25]. Wagner-Jauregg was a frequent participant at the scientific meetings and one of his earliest contributions was on experimental work on the function of the thyroid gland [15, 23]. He was a member of the board for several years before being appointed vice president of the College in 1919. He held this position until 1932, after which he served as honorary president. The pharmacologist Otto Loewi, winner of the Nobel Prize for Physiology or Medicine in 1936, also presented important findings at the College of Physicians [15]. Karl Frisch (1886–1982) and Konrad Lorenz (1903–1989), who were awarded the Nobel Prize in 1973 together with Nikolaas Tinbergen (1907–1988), were also associated with the College. Frisch, who was born in Vienna and educated in Vienna and Munich, was not only a corresponding member of the College since 1937 but also gave a lecture to the College in 1933, shortly after the Munich Zoological Institute was founded. Konrad Lorenz was awarded honorary membership and the Billroth Medal in 1984 [15, 22].^2^ Sigmund Freud (1856–1939) also delivered his seminal lecture, “On Male Hysteria”, at the College in 1886, although it was met with considerable opposition [28–30].

This most important era in the history of the College of Physicians came to an end when the National Socialists came to power in Austria. Much like Viennese medicine as a whole, it was never able to recover from the resulting brain drain. The aim of this project is to analyze the College of Physicians systematically in the historical context of National Socialism, with a focus on its social, institutional and scientific development in the pivotal years between 1930 and 1960.^3^

## Effects of the National Socialist takeover

Within the College of Physicians in Vienna, the Nazi takeover increasingly led to the discrimination of Jewish members, ultimately resulting in their withdrawal. In 1938 the College of Physicians had 890 members, 541 of whom were considered Jewish under the Nuremberg Laws.^4^ We documented a total of 620 victims of Nazi persecution. Of the 28 female members of the College of Physicians at this time, 26 were of Jewish origin. Membership had fallen sharply even before 1938, possibly due to the departure of Jewish members before the *Anschluss* [31].

Immediately after the *Anschluss*, the College of Physicians in Vienna began to be reorganized in line with the National Socialist regime [32]. Consequently, the majority of the functionaries, around one third of whom were of Jewish origin, were forced to resign. The important role of the “first librarian” and responsibility for the most important library in Viennese medicine was taken on by the medical historian and gynecologist Isidor Fischer (1868–1943) [33]. Board members included the internist Maximilian Weinberger (1875–1954), the gynecologist Carl Fleischmann (1859–1941), the ear, nose and throat specialist Hugo Frey (1873–1951), the surgeon Julius Schnitzler (1865–1939), Josef Thenen (1866–1949), a social medicine specialist and former president of the Vienna Medical Chamber and the ophthalmologist Ludwig Sallmann (1892–1975), all of Jewish origin. They all resigned from their posts a few days after the *Anschluss*. Wilhelm Knöpfelmacher (1866–1938), a pediatrician who would commit suicide shortly after the *Anschluss* [34], was elected one of the three chairmen of the scientific meetings and a member of the board on 25 February 1938. Due to the *Anschluss*, he was no longer able to fulfil these functions.^5^

## Exclusion of Jewish members

Following the *Anschluss*, Adolf Irtl (1867–1947) [35], long-standing administrator of the College of Physicians in Vienna, was appointed provisional head of its replacement organization, the Medical Society of Vienna (*Wiener Medizinische Gesellschaft*). In this position, the former in-house physician at the Vienna State Opera and the “Burgtheater” was responsible for implementing successive discriminatory measures and excluding Jewish members from the new organization. The old College of Physicians was finally dissolved on 14 October 1938, in accordance with the “Law on the Transfer and Integration of Associations, Organizations and Federations” (GBlÖ Nr. 136/1938) of 17 May 1938 and the “Decree of the Reich Governor on the Implementation of the Law” (GBlÖ Nr. 136/1938). The Medical Society of Vienna, established in February 1939, did not accept Jewish members according to its statutes.

### Adolf Irtl (1867–1947)

Like pediatrician Franz Hamburger (1874–1954), Adolf Irtl had been a member of the Corps Rhenania Heidelberg since student days. He had also been a member of the antisemitic Association of German Physicians (“Verein Deutscher Ärzte”) in 1904 and a founding member of the German Club (“Deutscher Klub”) in 1908. Following the *Anschluss*, he applied to join the National Socialist German Workers’ Party (NSDAP) local group *Wien-Stadtpark* and was listed as a party candidate from 1 March 1938. He also became a member of the National Socialist German Doctors’ League in 1938. The following was his political assessment as part of his admission to the NSDAP: “As early as 1919 he turned to the national idea and displayed an antisemitic attitude.” A letter signed by Otto Planner-Plan (1893–1975), *Gauärzteführer* (Gau Physicians’ Leader), attested to Irtl’s “impeccable National Socialist convictions”. In a self-description prepared for the personnel questionnaire for application for a provisional NSDAP membership card, he described himself as “a Transylvanian Saxon […] by nature always national, antisemitic and in favor of the national community”. Irtl’s application for NSDAP membership was rejected on the grounds of his late application and insufficient “activity for the movement during the prohibition period (*Verbotszeit*)” and deferred until the ban on new members was relaxed. He was finally admitted to the NSDAP in 1943.

In his position as provisional head of the College of Physicians, Irtl’s main concern was how to deal with its Jewish members. On 18 March 1938, the day Eiselsberg resigned as president, he queried the Berlin Medical Society [36] on “How did you deal with your members who were Jews and Jewish half-breeds when the NSDAP took power, and can members of your highly esteemed association still be Jewish now?”^6^

On 9 June 1938, a meeting convened by Irtl took place at Billrothhaus to discuss continuing the College in line with the new rulers. Those in attendance included Oskar Kauffmann (1898–1955), at the time the top Nazi party official in charge of physicians in Austria (*Landesärzteführer*), Max Warsow, Head of Department IV Ad with responsibility for restructuring medical associations and a representative of Albert Hoffmann (1907–1972), the Reich Commissioner tasked with taking control of organizations, clubs and associations in Vienna [37], Otto Planner-Plann^7^, head of the Vienna Nazi Party’s Office of Public Health and members of the Faculty of Medicine of the University of Vienna, including the internist Hans Eppinger jun. (1879–1946), the pediatrician Franz Hamburger and the ophthalmologist Karl David Lindner (1883–1961). The new dean Eduard Pernkopf (1888–1955) chaired the meeting.

It was decided that the Jewish members should continue to pay the membership fee for 1938, as the College was dependent on this income. At this meeting, Irtl said: “non-Aryans will no longer be members in the future. And since the medical association must continue to exist, if only for reasons of prestige, so that it does not cease to exist with the departure of the Jews, [I believe] that it should be maintained with the help of state subsidy.” Max Warsow replied: “As long as no new order has been established and as they still feel like members, the Jews shall confidently continue to pay. For reasons of public health, the current situation cannot be abruptly terminated, but must be changed organically.”^8^ By 21 July 1938, it was unclear whether the College of Physicians would be dissolved as an independent association, after new statutes had already been drawn up in which it was stated that “only Aryans according to the Nuremberg Laws can become members.”^9^

While Irtl often waived part of the membership fee for “Aryan doctors” who were in financial need, as can be seen from Adolf Irtl’s individual replies to petitioners, he rejected petitions of Jewish members who had been restricted in their medical activities by the loss of their health insurance license and their licence to practise medicine as well as the ban on practising medicine imposed on 30 September 1938. Irtl’s letters of reply were essentially all worded in the same way: “On behalf of Reich Commissioner Gauleiter Bürckel, I must vigorously collect the outstanding membership fees.” If fees were not paid, members were excluded and threatened with legal action.

**Fig. 1:**
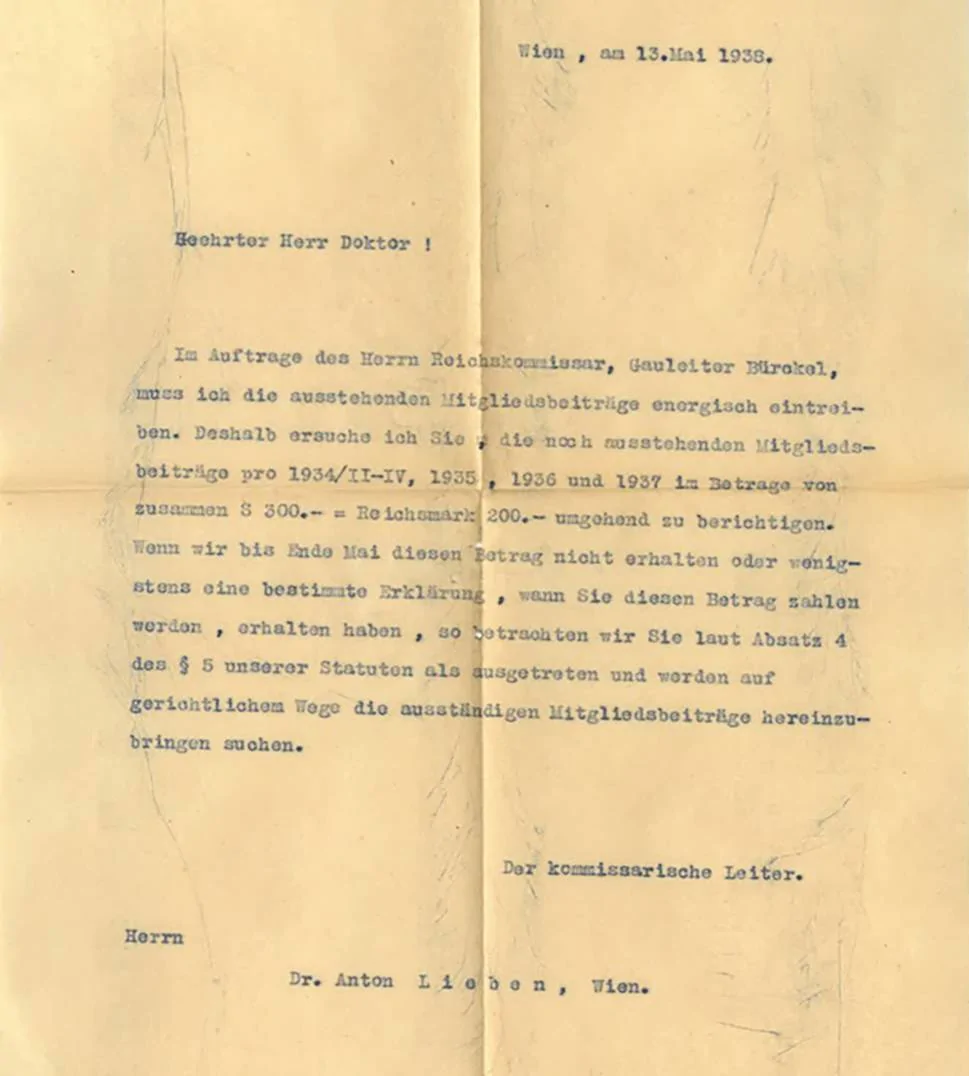
Letter from provisional director Adolf Irtl to Anton Lieben (1881–1939). (Source: AGDÄ, College of Physicians in Vienna (hereafter CPV), record cards)

According to the 1938 invoice book, around 350 “Jewish” members paid the “vigorously collected” membership fee for that year.^10^ Considerable sums were collected, as in the case of urologist Anton Lieben (see Fig. 1), who paid 300 Schilling (equivalent to 200 Reichsmark). The transition to excluding the Jewish members was ultimately seamless. According to § 3 of the statutes of the Medical Society of Vienna, only doctors who fulfilled the following criteria could be accepted as full members:

1. insofar as they meet the requirements of the Reich Citizenship Law,
2. persons of German blood living abroad,
3. foreigners, if they are German-friendly and recommended by the executive committee.”

Section 7 of the statutes stipulated that membership expired “upon expulsion from the Medical Chamber.” This meant that Jewish members would have been excluded from the College of Physicians due to the loss of their license to practice medicine [38], even without the establishment of a new National Socialist replacement organization [39].

## The victims within the College of Physicians in Vienna

So far, only a few of those excluded from the College of Physicians in Vienna have been honored in this context. Some of their fates are presented here as examples. Further information on the displaced persons that we have documented is available as part of the project “Memorial Book: expelled members of the College of Physicians in Vienna 1938”.^11^

Several resignation letters from Jewish members of the board of the College of Physicians were found in the College’s archive during research. On 16 March 1938, Carl Fleischmann resigned by stating the following to Irtl: “I kindly request that you relieve me of my position as a member of the board of directors of the College of Physicians”^12^. Julius Schnitzler, the younger brother of the author Arthur Schnitzler (1862–1931), announced his resignation on 15 March 1938: “Dear Mr President, please kindly note that I relinquish my position as a member of the board of directors of the College of Physicians in view of the changed circumstances […].”^13^

Isidor Fischer became the College of Physicians “first librarian” in 1921. Theodor Puschmann (1844–1899), Professor of the History of Medicine, had already held this prestigious position in the nineteenth century [15]. In the 1920s, Fischer created a card catalogue of the monographs and offprints in the library. At the time, he was responsible for the College’s extensive archive, which he restructured [20]. The College of Physicians archive was not only an organizational archive but also held an important collection of estates, manuscripts, literature, archival documents and pictures.^14^ Along with the Institute for the History of Medicine’s collection, largely sourced from the College’s archive [40, 41], which was built up by Max Neuburger (1868–1955), the College of Physicians owned the most important holdings on the history of medicine. In the run-up to the 100th anniversary of the College, Fischer wrote the *Festschrift* “History of the College of Physicians in Vienna: 1837–1937”, commissioned by the College’s board. It was published in 1938 without naming the author, for fear of complications due to his Jewish origins. In the preface to the book, dated the end of February 1938, the president of the College of Physicians, Eiselsberg, thanked the “honorary first librarian” without mentioning his name (Figs. 3 and 4) [15]. The Springer Publishing Company had already introduced the practice of not naming Jewish authors or publishers on the title page in National Socialist Germany. Although the Viennese publishing house was not directly affected by the regulations of the German Reich until the *Anschluss*, it became difficult for Springer to publish a book by a Jewish author intended for the German market in 1938. One reason for this was that as a German citizen, the owner of the publishing house, Ferdinand Springer Jr. (1881–1965), also had to comply with the regulations that applied to foreign companies in Germany [42]. Fischer managed to flee to London in autumn 1938 and declared his resignation from the College, of which he had been librarian for many years, on 29 September 1938 [20]. 15 His “History of the College of Physicians” remains the reference work to this day and is frequently cited [22, 43].

In 1930, Fischer had failed to obtain an associate professorship. He was accused of “chasing after” a gynecological practice for monetary reasons. Furthermore, it was claimed that he had sought his private lectureship and the desired title of titular professor for “advertising purposes” and out of “business acumen.”^16^ Such accusations were typical strategies employed by antisemitic networks to hinder the careers of Jewish scientists [3, 9, 44]. Fischer tried to defend himself by pointing out that he was unable to earn a regular income as a medical historian and therefore was forced to run a private gynecological practice [45]. 17.

The Fischer family’s flight was facilitated by medical historian Charles Singer (1876–1960) and, above all, by his wife Dorothea, who later also helped Neuburger to flee Austria at the last moment [46]. Singer played a significant role in assisting refugees fleeing Nazi persecution [47]. Fischer and his wife Marianne, née Baum (1880–1945), had been married since 1899. They had three children and initially found refuge in the UK with their son Leopold Johann (1900–1987), who by then was working as an engineer in London.

In England, Fischer continued his research into medical history, conducting research at the British Museum, the Royal College of Surgeons and the Royal Society of Medicine [48, 49]. He was also active within the Jewish Medical and Dental Association [46]. In 1931, Fischer published the book *Der Eigennamen in Krankheitsterminologie (Proper names in medical terminology)*, a dictionary of medical eponyms based on personal names. While in England he devoted much of his time to working on a supplementary volume on geographical eponyms (*Geographical Names in Medical Terminology*) [50]. He also published the eminent study “British Medicine and the old Viennese School” in the *Bulletin of the History of Medicine* [51], which introduced the history of Viennese medicine to an English-speaking audience. Another publication, “Jews in modern medicine” [51], reflects his involvement with Jewish medicine, which accompanied him throughout his career. Fischer lived in precarious circumstances. Not only did he suffer from the consequences of his escape and the lack of financial earning opportunities, but also from the 6‑month internment by the British authorities of his son Leopold as an “enemy alien” [48]. The family eventually moved to Bristol. It was there that he was diagnosed with Hodgkin’s lymphoma. He died in Bristol on 13 January 1943.^18^

**Fig. 2:**
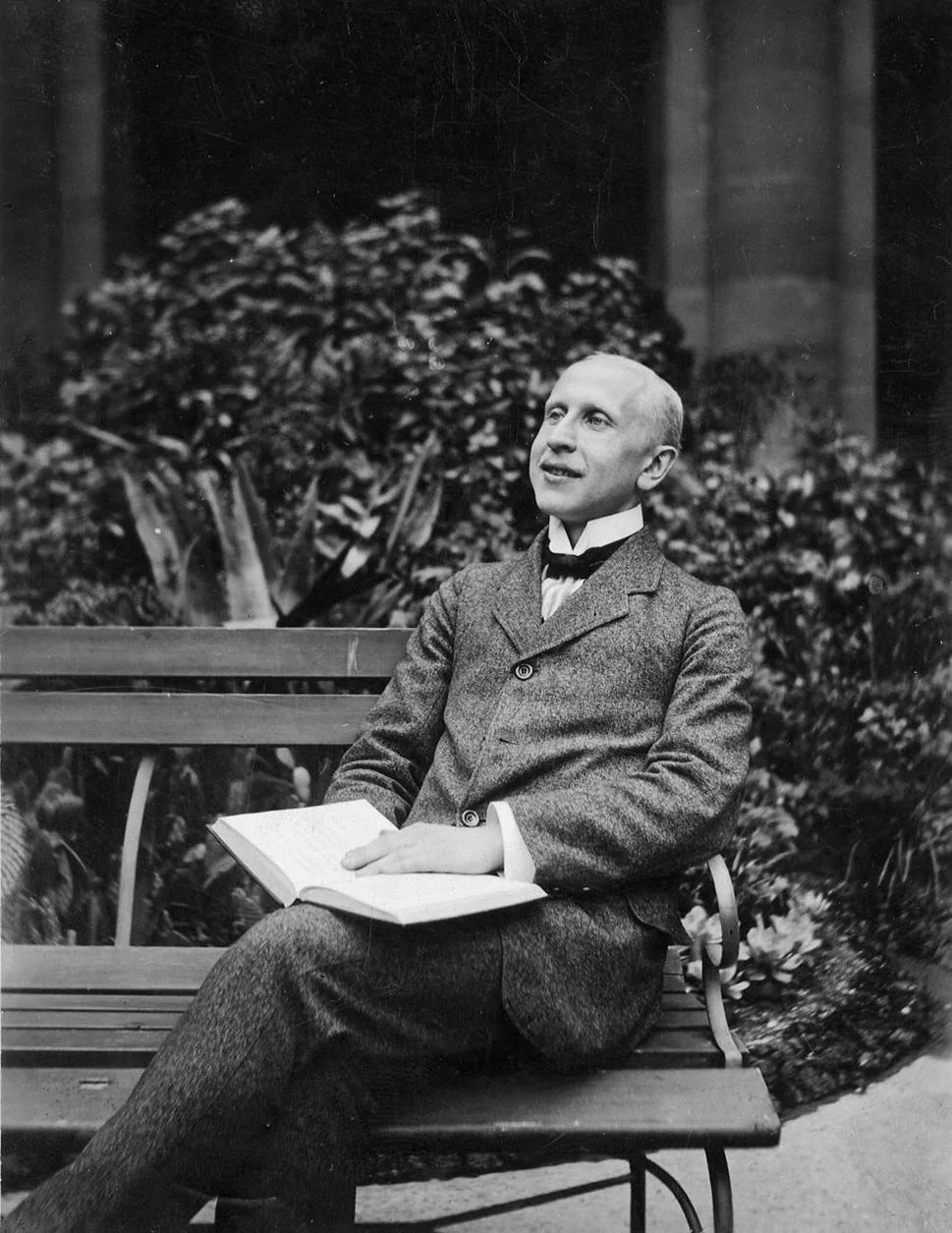
Isidor Fischer (1868–1943). Source: AUW, picture collection, 106.I.4071

While in London, Fischer was assisted by Dr. Hedwig Pollak, the former secretary of the College of Physicians, who had also been forced to flee. Pollak acted as Fischer’s assistant and closest collaborator [22, 48, 50]. She helped him work on his unfinished book *Geographical Names in Medical Terminology* and continued to edit it after his death [50]. A typescript, revised by Pollak and later by L.T. Norton, is now held by the Wellcome Collection.^19^

**Fig. 3:**
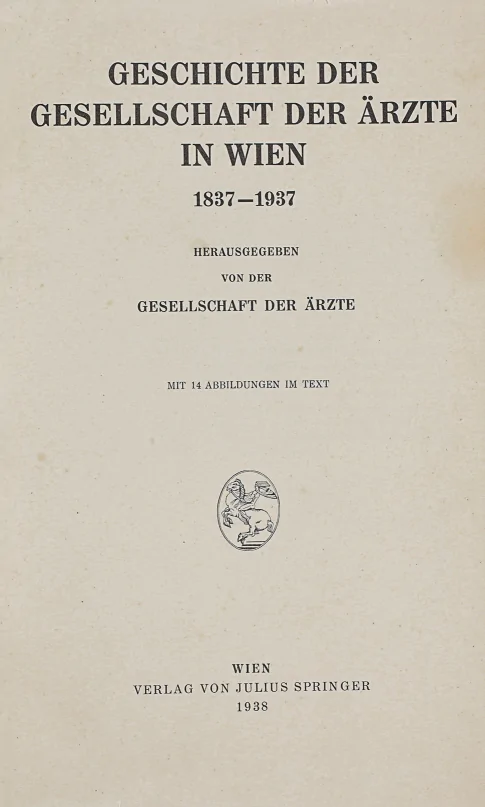
Title page of the anonymized book *History of the* *College of Physicians in Vienna: 1837–1937*

**Fig. 4:**
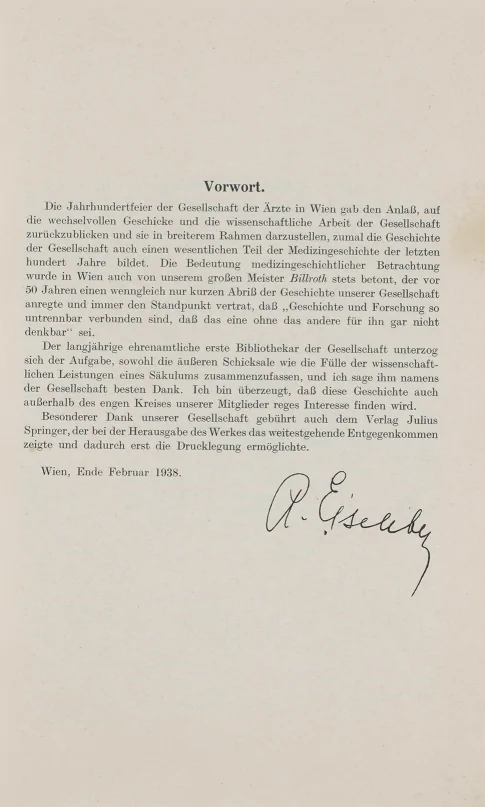
Preface of the anonymized book *History of the College of Physicians in Vienna: 1837–1937*

**Fig. 5:**
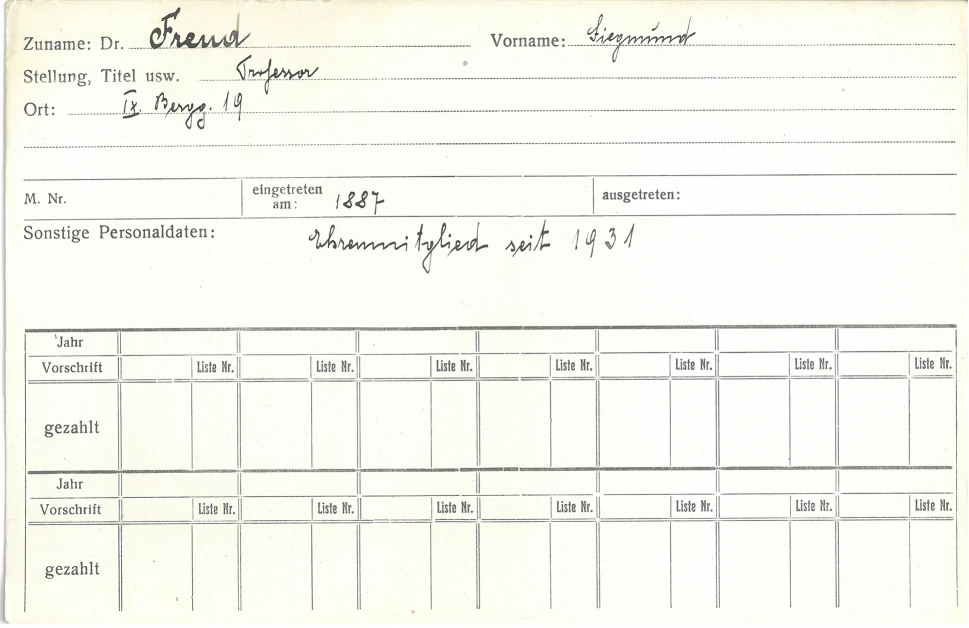
Sigmund Freud’s record card. As honorary member, he did not have to pay membership fees. (Source: AGDÄ, CPV, record cards)

Maximilian Weinberger, a lung and heart disease specialist, was one of the last Jewish doctors to flee Austria. Although the Second World War had already broken out, he was able to leave Austria in 1941 and flee to New York [52, 53]. Julius Schnitzler died in Vienna before the outbreak of the Second World War in June 1939. Carl Fleischmann managed to flee to London in September 1938 [54]. Josef Thenen and his second wife fled to Romania in October 1939, just in time. Thenen’s first wife, Hermine Doctor, was murdered by the National Socialists in 1942 [55]. Ludwig Sallmann [56] and Hugo Frey [57] also managed to escape in 1939. Sigmund Freud, who had been a member since 1887 as well as honorary member since 1931, fled to England with his wife in spring 1939 ([28]; Fig. 5).

**Fig. 6:**
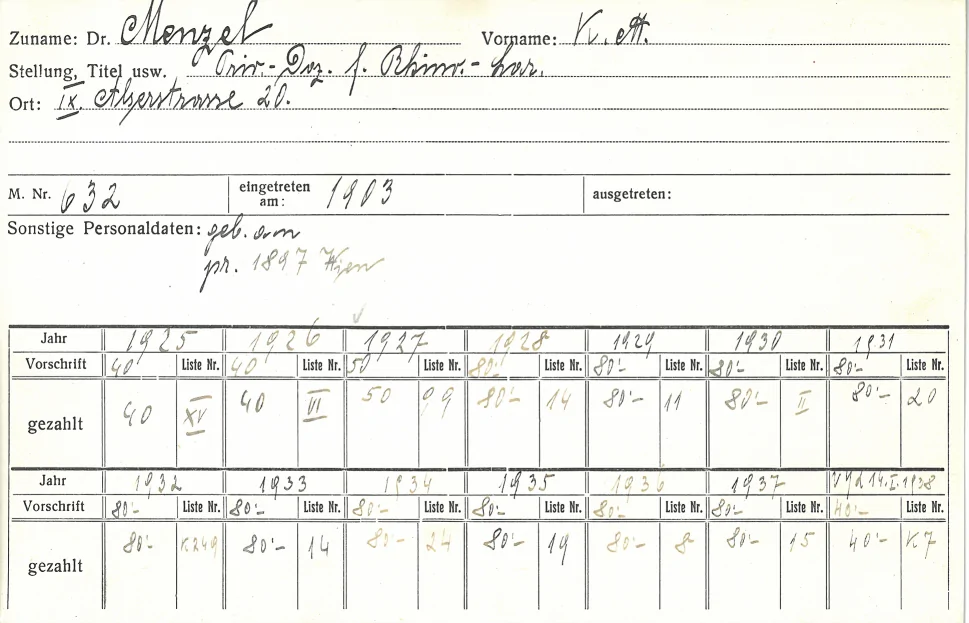
Record card of Karl Moriz Menzel

**Fig. 7:**
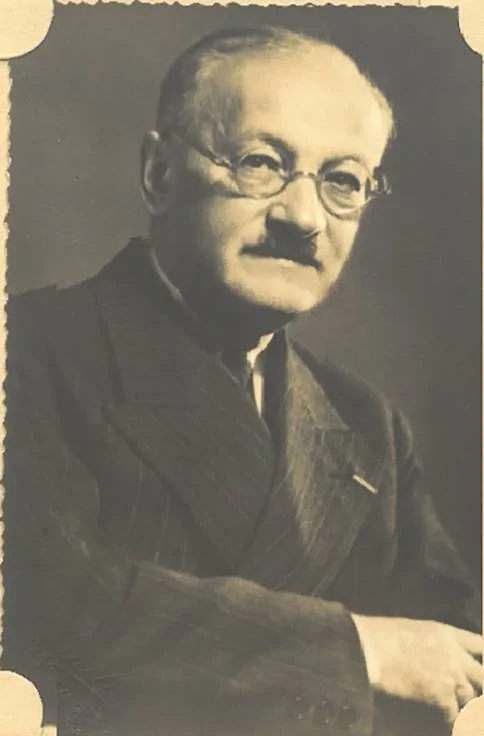
Karl Moriz Menzel (1873–1944). (Source: AGDÄ, CPV, record cards)

One of the displaced members was the laryngorhinootologist Karl Moriz Menzel (1873–1944). Menzel and his family managed to flee to Brussels in 1938 but were forced to stay there after the Nazi invasion, despite repeated attempts to find a safe haven. Menzel died in 1944, after which his family emigrated to the USA having persevered until the end of the war ([31]; Figs. 6 and 7).

**Fig. 8:**
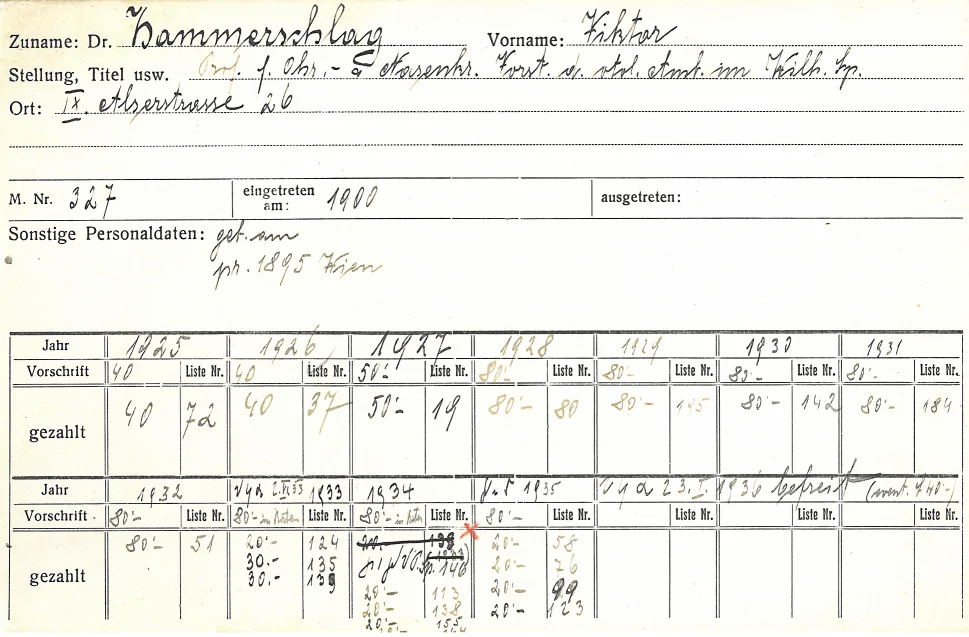
Record card of Viktor Hammerschlag. Source: AGDÄ, CPV, record cards

**Fig. 9:**
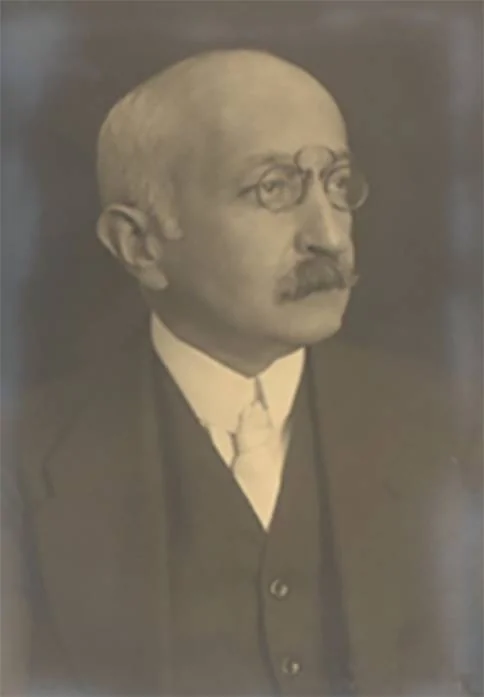
Viktor Hammerschlag (1870–1943). Source: Josephinum—Medical University of Vienna, AT-MUW-FO-001924-0001-0001

Viktor Hammerschlag (1870–1943), Associate Professor of Otology at the Medical Faculty of the University of Vienna from 1911, was one of the College’s members murdered in the Holocaust. He had been a member since 1900 (see picture of his record card; Fig. 8). Hammerschlag and his wife Hedwig were deported to the Theresienstadt concentration camp in 1942 and murdered on 16 May 1943. One of their two sons, the cabaret artist Peter Hammerschlag (1902–1942), was also murdered in Auschwitz. Their other son, Valentin Hammerschlag (1909–1975), managed to flee to Argentina in time ([58]; Figs. 8 and 9).

In the course of the project “Memorial Book: expelled members of the College of Physicians in Vienna in 1938”, we investigated the fate of each victim. A virtual memorial book containing a short biography and their names has been available since 3 December 2025 (https://www.billrothhaus.at/index.php?option=com_sf_memorialplaque&task=showIntro).

Among the members of the College of Physicians in Vienna, we have documented 44 victims who were deported and died in (or en route to) concentration or extermination camps, and 8 persons who committed suicide due to the experienced persecution. Of the victims six died in “Sammelwohnungen”.

## Remembrance after the Second World War

After the war, the College of Physicians sought to commemorate the victims of National Socialism. At the first provisional board meeting after the Second World War on 20 June 1945, dermatologist Leopold Arzt (1883–1955), the former “Second Secretary” and only member of the Board of the College of Physicians before the *Anschluss* present in Vienna at this time, requested that “a special solemn memorial meeting be held at the beginning of the winter semester for the members who have died since 1938.” This was unanimously approved [59]. On 19 October 1945, the newly elected Board decided to open the first scientific meeting of the reinstated College of Physicians on 2 November 1945 with a “memorial service”.^20^ The choice for delivering the commemorative speech fell on forensic pathologist Friedrich Reuter (1875–1959), who, like Arzt, had himself been persecuted during the National Socialist era due to his proximity to the Dollfuß-Schuschnigg dictatorship (the authoritarian government that was established in Austria from 1933/34 until the country’s Anschluss in March 1938, based on corporatist and fascist ideas). After the *Anschluss*, Reuter was forced to retire permanently as an associate professor of forensic medicine. He was replaced by Philipp Schneider (1896–1954) as director of the institute. Schneider had also been active in the Medical Society of Vienna. After the Second World War, Reuter, a friend of Arzt, was able to resume his position as institute director and became Vienna’s City Councillor for Public Health for the conservative Austrian People’s Party (ÖVP).^21^ Initially, psychiatrist and neurologist Otto Kauders (1893–1949) had also been shortlisted for delivering the commemorative speech.^22^

Reuter’s speech, dedicated to the memory of those who died between 1938 and 1945, began with the following words: “Today, we wish to dedicate an hour of faithful remembrance to our colleagues who were cruelly torn from our ranks between 1938 and 1945. Many of them fell victim to the terrible struggle in the greatest battle in human history, some while carrying out their professional duties in hospitals, others in concentration camps, branded as enemies of the people and as criminals. Some died of natural causes after a busy life dedicated to the welfare of their fellow human beings” [60].

The commemoration activities continued. On 22 March 1946, Leopold Arzt, himself arrested after the *Anschluss* for 6 weeks due to his association with the Dollfuß-Schuschnigg dictatorship and forced to retire, gave a memorial speech at the first annual general meeting after the Second World War. In his speech, he focused on the fate of the Jewish otolaryngologist Heinrich von Neumann (1873–1939), representing all those expelled in 1938 [61].

Upon this speech, an inscription was added to the memorial plaque in early 1946, located in the stairwell of the College of Physicians (the Billrothhaus), which originally had been dedicated to the College members who had died in the First World War. According to Arzt, the initiative for this came from “politically or racially victimized members of the teaching staff of the Vienna Medical Faculty”, who approached the board of the College of Physicians. Their plan was to have a prominent physician give a commemorative speech at the annual general meeting every year [61]; however, this idea was never realized. The exception was a memorial speech delivered by Viktor Frankl (1905–1997), who would soon come to worldwide fame, at the annual general meeting on 25 March 1949.

Frankl trained as specialist in neurology and psychiatry and from 1933–1937 was head of the “suicide pavilion” (“Selbstmörderpavillon”) at the University Psychiatric Clinic. In 1937, he opened a private practice which he was forced to close after the *Anschluss*. In 1939, he allowed his exit visa to America to expire so that he could stay in Vienna with his parents. From 1940–1942, he was head of the neurology department at the Rothschild Hospital. On 25 September 1942 Frankl was deported to Theresienstadt with his wife and parents. He survived a total of four concentration camps, including Auschwitz, to which he was transferred from Theresienstadt on 19 October 1944, where his brother was murdered. He was the sole survivor of the Holocaust in his family. On 5 March 1945, he was sent to a subcamp of Dachau concentration camp, where he was liberated by the US Army on 27 April 1945. His book “… Yes to Life: In Spite of Everything: A Psychologist Experiences the Concentration Camp” was published in 1946 [62]. Frankl’s speech at the College of Physicians on 25 March 1949 resounded particularly tragic, as he himself had experienced the fate and murder of the seven doctors he described as concentration camp detainees [60, 63] (the speech is reproduced in full in the appendix).

“In memoriam … in commemoration … ‘What is mankind that you are mindful of them, human beings that you care for them?’ This is a question the psalmist puts to God. Let us pose this question towards ourselves here today and ask: what were our deceased colleagues that we remember them on this day? Well, how they had lived and died among you from 1938 to 1945, how they had lived and perished in prison and in exile, you do know or you have been told on previous occasions. It is my duty to bear witness before you to how Viennese doctors languished and expired in the concentration camps; to bear witness to true doctors—who lived as doctors and died as doctors; to true doctors, who could not bear to see others suffer, who could not allow others to suffer, yet forced themselves to suffer, learning, understanding real suffering—an upright suffering” [63].

„Was waren die toten Kollegen, daß wir an diesem Tage ihrer gedenken? Nun, wie sie von 1938 bis 1945 in Ihrer Mitte, wie sie im Kerker und wie sie in der Verbannung gelebt und gestorben, das wissen Sie oder hat man Ihnen aus früheren Anlässen bereits berichtet. Meine Aufgabe ist es, vor Ihnen Zeugnis abzulegen, wie Wiener Aerzte in den Konzentrationslagern, geschmachtet und geendet haben; Zeugnis abzulegen von wahren Aerzten – die als Aerzte gelebt haben und als Aerzte gestorben sind; von wahren Aerzten, die Andere nicht leiden sehen, nicht leiden lassen konnten, selber aber zu leiden verstanden, selber das rechte Leiden zu leiden wußten – das aufrechte Leiden!“

## The memorial plaque at the Billrothhaus

Both Leopold Arzt’s memorial speech at the first annual general meeting after the Second World War as well as the memorial plaque contain a formulation inappropriate from today’s perspective, namely that the intention was to commemorate “those who died during the years of occupation as victims of their convictions for faith and fatherland.”^23^ There was no explicit acknowledgement of the largest victim group, physicians persecuted because of their Jewish family background [61].

In the 1920s, Leopold Arzt held a powerful position within the Vienna Medical Faculty and had considerable influence over important decisions [64].^24^ He was dean of the Medical Faculty between 1927 and 1930, a senator at the Medical Faculty between 1933 and 1936, and rector of the University of Vienna in 1936/1937. He was a member of the *Bundeskulturrat* and the *Bundestag* from 1934–1938 [9]. The *Bundestag* was a legislative body established under Austria’s Austrofascist system. Following the elimination of the National Council and the Constitutional Court, it formally confirmed the federal government’s bills. According to the memoir of Julius Wagner-Jauregg, Arzt was the most powerful professor within the Faculty of Medicine from the mid-1920s to the *Anschluss*, he dominated and directed the faculty [64]. He prevented the habilitation and appointment of Jewish, liberal, socialist and communist academics [9, 65], not least through his participation in secret networks such as the “*Deutsche Gemeinschaft*.”^25^

**Fig. 10:**
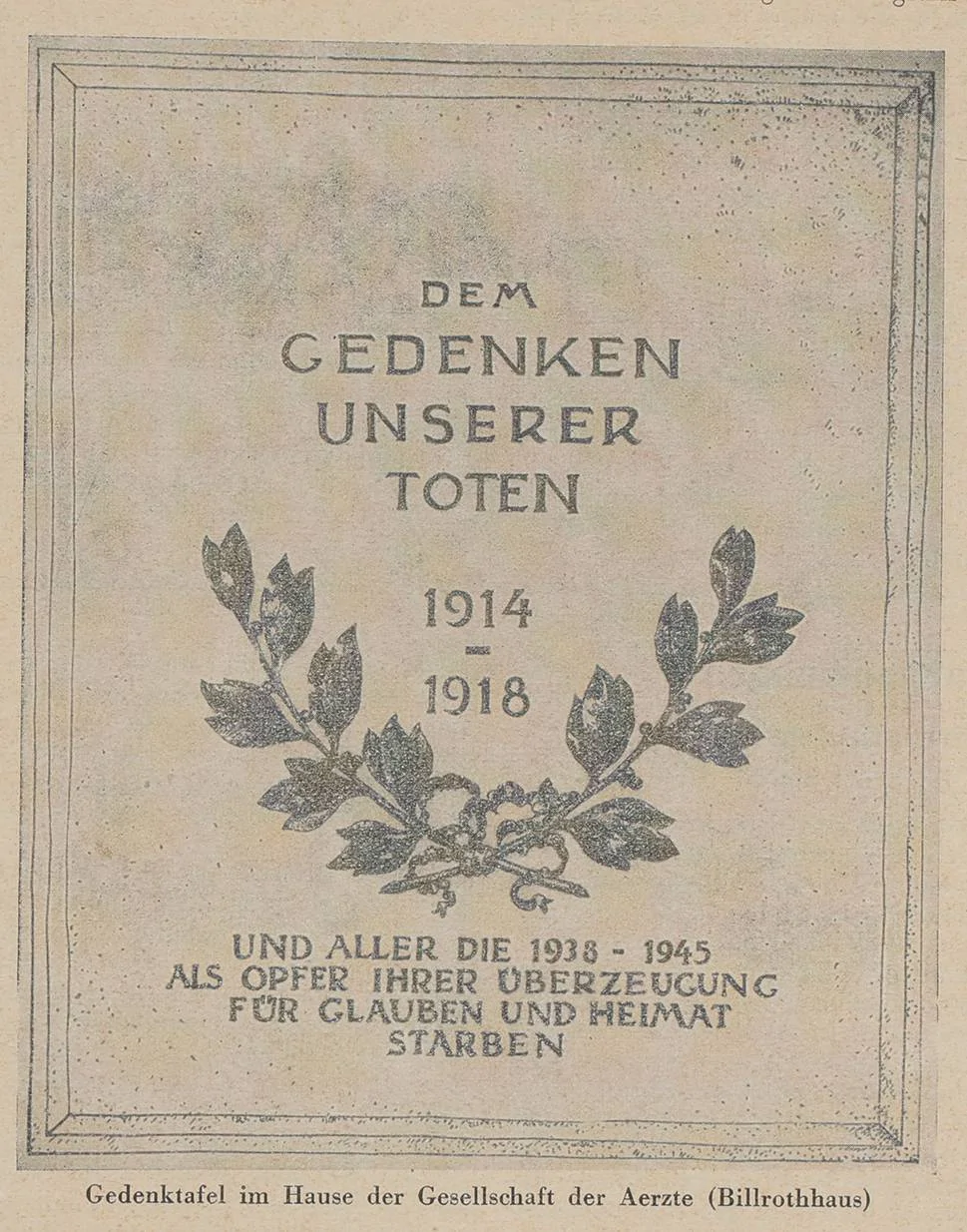
The original memorial plaque erected in 1927, with the addition from 1946. A photograph of this augmented plaque was published alongside Leopold Arzt’s memorial speech in the *Wiener klinische Wochenschrift* in 1946

**Fig. 11:**
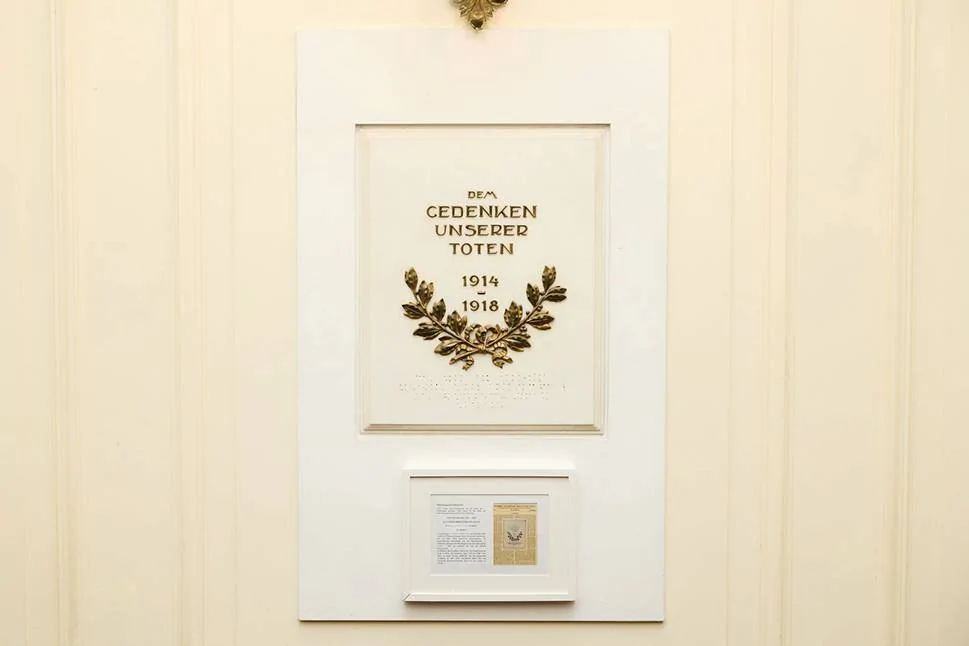
The memorial plaque in its current state (Photograph by S. Burghart)

**Fig. 12:**
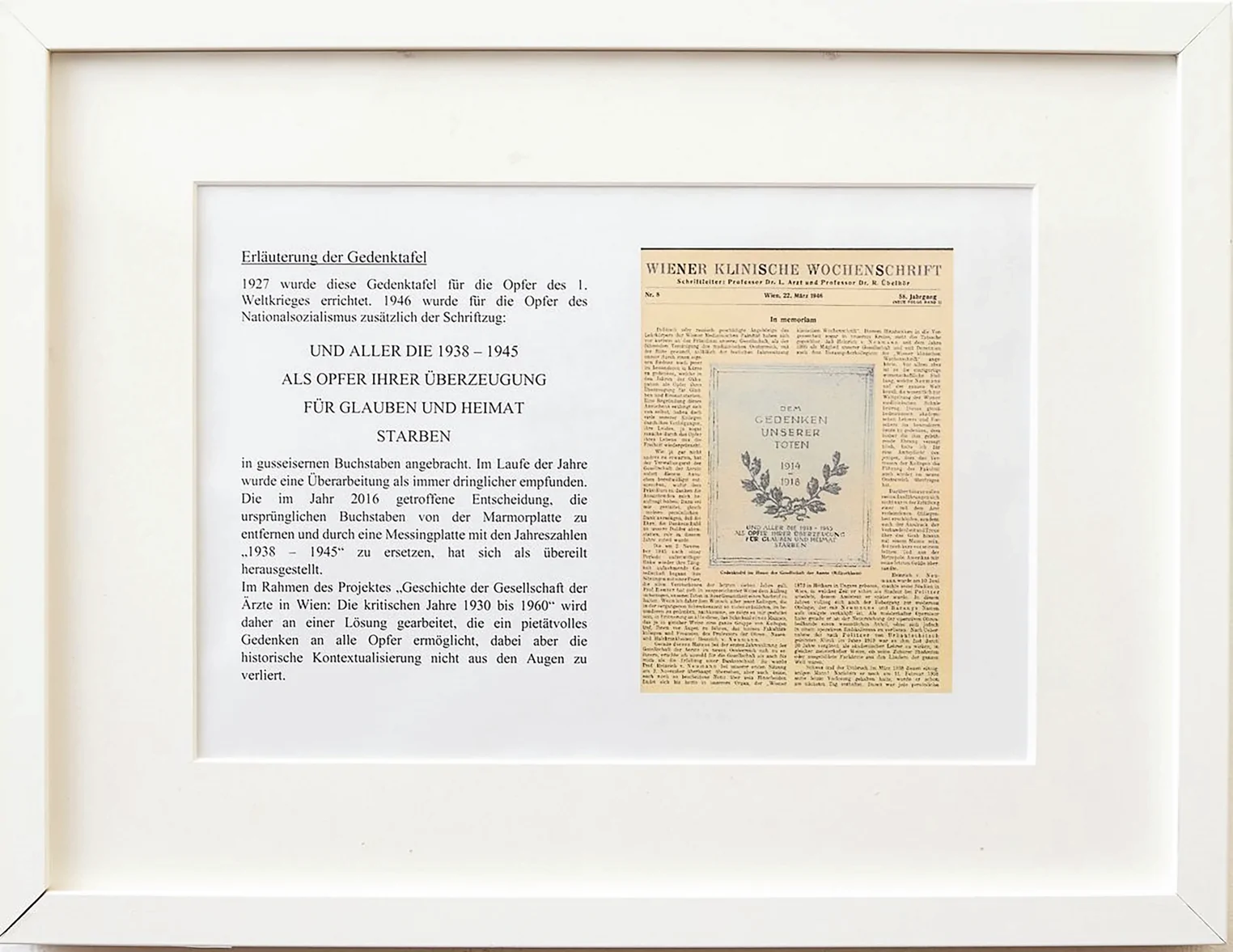
An added explanatory panel puts the memorial plaque in historical context (Photograph by S. Burghart)

Not only did Arzt take charge of the rebuilding of the College of Physicians in the summer of 1945 he also served as Dean of the Faculty of Medicine in the summer semester of 1945 and in the 1945/1946 academic year. He thus represents a continuity from the interwar period into the aftermath of the Second World War, representing the Catholic elites that had dominated before the *Anschluss*, and the universities’ political orientation they had shaped ([66]; Figs. 10, 11 and 12).

In 2016, the Presidium of the College of Physicians decided to remove the cast-iron letters of the inscription from the monumental plaque and replace them with a brass plaque bearing the text “1938–1945”. From today’s perspective, this decision was somewhat hasty, as it failed to account for the plaque’s historical significance. In response to this, the decision was made to remove the brass plaque. From a conservation point of view, it is no longer possible to affix the original letters and restore the plaque to its 1946 condition. This facilitated the decision to leave the plaque in its current state (see Fig. 11) and contextualize it for historical reflection. The project “History of the College of Physicians in Vienna: The Critical Years 1930–1960”, as initiated by the College of Physicians in Vienna, has succeeded in identifying the victims of the National Socialist expulsion. It was decided that the most dignified and adequate commemoration would take the form of a virtual memorial book depicting all the names and causes for persecution. In addition, a small display case will be installed to provide context for the memorial plaque and to establish a link to the virtual memorial book. In the meantime, a provisional accompanying text has been added. The project, “Memorial Book: Expelled Members of the College of Physicians in Vienna 1938” aims to create a lasting tribute to all those affected by the National Socialist expulsion. A total of 620 individuals have been documented; however, the memorial book is an ongoing project that will be continuously supplemented with further research on individual biographies. The College of Physicians in Vienna and the project team view the memorial book as a symbolic initiative and commemoration for present and future generations.

### Link

Appendix [63]

## Appendix

### In memoriam

Memorial address for the members of the College who died between 1938 and 1945, delivered at the annual meeting of the College of Physicians in Vienna on 25 March 1949. Viktor Frankl^26^

In memoriam … in commemoration … “*What is mankind* that you are mindful of them, human beings that you care for them?” This is a question the psalmist puts to God. Let us pose this question towards ourselves here today and ask: What were our deceased colleagues that we remember them on this day? Well, how they had lived and died among you from 1938 to 1945, how they had lived and perished in prison and in exile, you do know or you have been told on previous occasions. It is my duty to bear witness before you to how Viennese doctors languished and expired in the concentration camps; to bear witness to true doctors – who lived as doctors and died as doctors; to true doctors, who could not bear to see others suffer, who could not allow others to suffer, yet forced themselves to suffer, learning, understanding real suffering — an upright suffering!

It was in the summer of 1942. Everywhere, people were being deported, including doctors. One evening at Praterstern, I met a young dermatologist. We talked about being a doctor in those times, about a doctor’s mission in those times. And we came to talk about Albert Schweitzer, the jungle doctor of Lambarene, and our admiration for him. And then we talked about how we really had no reason to complain about a lack of opportunity to emulate this exemplary doctor and human being: We certainly had plenty of opportunities to provide medical assistance in a similar way to him under the most unfavourable circumstances imaginable. We certainly wouldn’t have to travel to the African jungle. We talked about all of this then, and promised each other that evening that we would recognise such an opportunity if the day came when we too were deported. Soon after, the day had come. Our young colleague, however, wasn’t granted much time to take advantage of the opportunity she had seen in her deportation based on her medical ethics: shortly after her arrival at the camp, she contracted typhus. A few weeks later, she was dead. Her name was Dr. Gisa Gerbel. We commemorate her …

Then there was a doctor for the poor from the sixteenth district – known in Vienna as “the Angel of Ottakring”; a true Viennese character – a man who, even in the camp, enthused about nothing else but reunion celebrations at a Viennese Heuriger [wine tavern], and then, with a blissful gaze and tears in his eyes, would sing the Heuriger song: “Only when it will be over ….” That was the Angel of Ottakring; yet who would protect him, who would be his guardian angel, back then, in Auschwitz, when he was directed away from the station to the left side before my very eyes – which meant: straight into the gas chamber? That was the Angel of Ottakring. His name was Dr. Plautus. We commemorate him …

Then there was Dr. Lamberg – the son of the first chief physician of the world-famous Vienna Voluntary Rescue Society – well known to all students of first aid from his textbook. Dr. Lamberg was a man of the world in both appearance and demeanour. Anyone who has ever seen this magnificent man knows it. Well, I also saw him when he was dying in a halfway underground barrack, amongst dozens of half-starved figures closely piled together; and his last request to me was to move the corpse lying next to and half on top of him a little to the side … That was former man of the world Dr. Lamberg, one of the few camp mates with whom one could have philosophical and religious conversations even during hardest labour on railway tracks in a snowstorm. We commemorate him …

Then there was Dr. Martha Rappaport, my former assistant at the Rothschild Hospital in Vienna, but also once assistant doctor under Wagner-Jauregg. A woman with a heart that could not see anyone cry without being moved to tears herself. Who cried for her when she was deported? – This was Frau Dr. Rappaport. We commemorate her …

Then there were, from the same hospital, a young surgeon, Dr. Paul Fürst, and another physician, Dr. Ernst Rosenberg. Both of them I had a chance to speak with shortly before they died in the camp. And there was not a single word of hatred in their last words – only words of longing came from their lips – and words of forgiveness; for what they hated and what we hate are never people – people must be forgiven. What they hated was solely the system, which brought guilt to some and death to others.

I have mentioned few names, and not in order of scientific rank. I speak of individuals but mean each and all of them together. The few must stand for the many, for the many could not be contained in a chronicle written by human hand. However, they are not in need of chronicles, nor demand they commemorative stones; for *every deed is its own monument* – evermore imperishable than one merely the work of our hands. For a person’s deed cannot be undone; what they accomplished cannot be eliminated. Thus, *it is not true *that *theirs is irretrievably lost in foretime —* rather it is *indelibly treasured* in that past!

It is true that the medical profession was desecrated in those years. But it is equally true that it was also held in high esteem in those same years. Some doctors experimented on the dying in the camps; yet there were others who experimented on themselves. I remember a neurologist from Berlin with whom I had many a nightly conversation in the sombre barracks, such as about current problems in modern psychotherapy. When he died in the camp, he documented the experience of his last hours in form of a written self-testimony.

Altogether, the experience of the concentration camp was one big experiment, a veritable *experimentum crucis*. Our dead colleagues endured it honourably. They proved to us that human beings have it in their power *to remain human even under the most adverse and undeserving conditions* , true humans and true doctors. What brings honour to those who proved this shall be our lesson, teaching us what humans are and what they can be.

*Now, what is a human?* We have come to know the human as perhaps no generation before us; we have come to *know* the human in the camp , in the camp, where everything unessential had melted away from each one; where everything one had held ceased to exist: money, might, fame, fortune; where merely remained what a human cannot “have” but must “be”: what remained was mankind’s self, seared by pain and scorched by suffering, was it molten to its essence, its bare humanity.

*So, what is a human?* We ask again. – A human is a being who always *decides* what to be. A being who harbors within themselves the choice of descending into the lowlands of the beast or of soaring to a saintly life. Man is the being who invented the gas chambers after all; then again it was human beings entering those very gas chambers in upright posture, reciting the Lord’s Prayer or the Kaddish.

*That, then, is a human.* By which we also know the answer to our query at the beginning: what is mankind that we are mindful of them, *human beings that we care for them?* “Human is a reed,” Pascal said, “– yet a reed that thinks!” And this thinking, this consciousness, this sense of responsibility is what constitutes human dignity, an individual’s dignity. It is always good and proper up to *the individual* alone to trample it under foot, or to cherish it. Just as one constitutes a human’s personal merit, so the other leaves him guilty. Whereby *there can only be personal* guilt! There must never be a question raised of collective guilt! Certainly, there is personal guilt of a person who may have “done nothing” but who has failed to do something; failed to act from fear for self or trepidation for loved ones. Yet anyone who comes to reproach such person of “cowardice” must firstly prove themselves a hero in that same situation.

Wouldn’t it be better, after all, to judge others not too harshly? Paul Valéry once said: “Si nous jugeons et accusons, le fonds n’est pas atteint”, as long as we judge and accuse, we haven’t reached the bedrock. Hence, not only, must we commemorate the dead, but forgive, also, the living. Just as we reach out to the dead, across all graves, we shall reach for the living, across all hatred. So when we utter: Honour be to the dead, let us append: and peace to all the living *who are of good will*.

### In memoriam

Gedenkrede für die in den Jahren 1938–1945 verstorbenen Mitglieder der Gesellschaft, gehalten in der Jahressitzung der Gesellschaft der Aerzte in Wien am 25. März 1949

Viktor Frankl^27^

In memoriam … Zum Gedenken … „*Was ist der Mensch*, daß Du seiner gedenkst?“ So lautet eine Frage, die der Psalmist an Gott richtet. Lassen Sie uns diese Frage hier und heute an uns selber richten und fragen: Was waren die toten Kollegen, daß wir an diesem Tage ihrer gedenken? Nun, wie sie von 1938 bis 1945 in Ihrer Mitte, wie sie im Kerker und wie sie in der Verbannung gelebt und gestorben, das wissen Sie oder hat man Ihnen aus früheren Anlässen bereits berichtet. Meine Aufgabe ist es, vor Ihnen Zeugnis abzulegen, wie Wiener Aerzte in den Konzentrationslagern geschmachtet und geendet haben; Zeugnis abzulegen von wahren Aerzten – die als Aerzte gelebt haben und als Aerzte gestorben sind; von wahren Aerzten, die Andere nicht leiden sehen, nicht leiden lassen konnten, selber aber zu leiden verstanden, selber das rechte Leiden zu leiden wußten – das aufrechte Leiden!

Es war im Sommer 1942. Allenthalben wurden Menschen, wurden unter ihnen Aerzte deportiert. Da traf ich eines Abends am Praterstern eine junge Dermatologin. Wir sprachen vom Arztsein in dieser Zeit, vom Auftrag des Arztes in dieser Zeit. Und wir kamen auf Albert Schweitzer zu sprechen, den Urwalddoktor von Lambarene, und auf unsere Bewunderung für ihn. Und dann sprachen wir davon, daß wir uns wohl nicht zu beklagen hätten über einen Mangel an Gelegenheit, diesem vorbildlichen Arzt und Menschen nachzueifern: Wir hatten wahrlich Chancen genug, gleich ihm unter denkbar ungünstigen Umständen ärztliche Hilfe zu leisten. Wir hätten wahrlich nicht erst in den afrikanischen Urwald zu reisen gebraucht. Von alledem sprachen wir damals, und wir versprachen einander an diesem Abend, eine solche Chance darin zu sehen, wenn der Tag kommen sollte, da auch wir deportiert würden. Um weniges später war dieser Tag gekommen. Allein, der jungen Kollegin war nicht viel Zeit gelassen, die Chance zu nützen, die sie aus ihrem ärztlichen Ethos heraus in der Deportierung gesehen hatte: Kurze Zeit nach ihrer Ankunft im Lager zog sie sich eine Typhusinfektion zu. Wenige Wochen später war sie tot. Ihr Name ist Dr. Gisa Gerbel. Wir gedenken ihrer …

Dann war da ein Armenarzt aus dem sechzehnten Bezirk – nicht anders bekannt in Wien als unter dem Namen „der Engel von Ottakring“; eine Urwiener Type – ein Mann, der noch im Lager von nichts anderem schwärmte als von Wiedersehensfeiern bei einem Wiener Heurigen, und dann mit seligen Blicken und Tränen in den Augen das Heurigenlied anstimmen konnte: „Erst wenn’s aus wird sein …“ Das war der Engel von Ottakring; aber wer hat ihn selbst beschützt, gleich einem Schutzengel – damals, als er in Auschwitz vor meinen Augen vom Bahnhof weg auf die linke Seite dirigiert wurde – und das hieß: direkt in die Gaskammer? … Das war der Engel von Ottakring. Sein Name Dr. Plautus. Wir gedenken seiner …

Dann war da Dr. Lamberg – der Sohn des ersten Chefarztes der weltberühmten Wiener freiwilligen Rettungsgesellschaft –, allen Erste-Hilfe-Schülern wohlbekannt von seinem Lehrbuch her; Dr. Lamberg war ein Weltmann im Aussehen wie im Auftreten. Das weiß jeder, der diesen prächtigen Menschen jemals gesehen hat. Nun, ich habe ihn auch gesehen, als er in einer halb unterirdischen Baracke im Sterben lag, inmitten Dutzender, eng nebeneinander liegender, halbverhungerter Gestalten; und seine letzte Bitte an mich war: die Leiche, die neben und halb auf ihm lag, doch ein wenig beiseite zu rücken … Das war der einstige Weltmann Dr. Lamberg – einer der wenigen Lagerkameraden, mit denen man selbst noch bei der schwersten Arbeit an Bahngeleisen im Schneesturm philosophische und religiöse Gespräche führen konnte. Wir gedenken seiner ….

Dann war da Frau Dr. Martha Rappaport, meine einstige Assistentin am Wiener Rothschild-Spital, aber auch schon Assistenzärztin einstmals unter Wagner-Jauregg. Eine Frau mit einem Herzen, das niemanden weinen sehen konnte, ohne selber zu Tränen gerührt zu sein. Wer hat um sie geweint, als sie deportiert wurde? – Das war Frau Dr. Rappaport. Wir gedenken ihrer …

Dann waren da, vom gleichen Spital, ein junger Chirurg, Dr. Paul Fürst, und ein weiterer Arzt, Dr. Ernst Rosenberg. Beide konnte ich im Lager sprechen, kurz bevor sie dort starben. Und es war in ihren letzten Worten kein einziges Wort des Hasses – nur Worte der Sehnsucht kamen über ihre Lippen – und Worte des Verzeihens; denn was sie haßten und was wir hassen, sind niemals Menschen – Menschen muß man verzeihen können; was sie haßten, war nur das System – das die einen in Schuld brachte – und den andern den Tod brachte.

Ich habe wenige Namen genannt, und die nicht dem wissenschaftlichen Rang nach; ich spreche von Einzelnen, aber ich meine alle mitsammen. Die Wenigen aber müssen für die Vielen stehen: denn die Vielen könnte keine Chronik fassen, die von Menschenhand aufgezeichnet würde. Aber sie bedürfen auch gar nicht einer Chronik, sie bedürfen auch nicht eines Gedenksteins; denn *jede Tat ist ihr eigenes Denkmal* – unvergänglicher als eines, das bloß unserer Hände Werk ist. Denn die Tat eines Menschen läßt sich nicht ungeschehen machen; was er getan, läßt sich nicht aus der Welt schaffen. Und *es ist* *nicht wahr, *daß *es in der Vergangenheit unwiederbringlich verloren* sei, sondern in der Vergangenheit ist es *untilgbar geborgen!*

Es ist richtig: In jenen Jahren wurde das Arzttum geschändet. Aber ebenso wahr ist, daß es in jenen Jahren auch hochgehalten wurde. Die einen Aerzte haben in den Lagern an Todgeweihten experimentiert; aber andere gab es, die hier an sich selbst experimentiert haben, und ich erinnere mich eines Berliner Nervenarztes, mit dem ich in der finsteren Baracke so manche nächtliche Zwiesprache gehalten hatte, etwa über aktuelle Probleme der modernen Psychotherapie; als er im Lager starb, hielt er das Erlebnis der letzten Stunden in Form einer Selbstschilderung schriftlich fest.

Ueberhaupt war das Erlebnis des Konzentrationslagers ein einziges großes Experiment – ein wahres *Experimentum crucis*. Unsere toten Kollegen haben es ehrenvoll bestanden. Sie haben uns bewiesen, daß der Mensch es in der Hand hat, *auch unter den ungünstigsten, den unwürdigsten Bedingungen noch Mensch zu bleiben* – wahrer Mensch und wahrer Arzt. Was denen, die diesen Beweis erbrachten, zur Ehre gereicht, soll aber uns eine Lehre sein, soll uns lehren, was der Mensch ist und was er sein kann.

*Was also ist der Mensch?* Wir haben ihn kennengelernt, wie vielleicht noch keine Generation vor uns; wir haben ihn *kennen*gelernt im Lager – im Lager, wo alles Unwesentliche vom Menschen weggeschmolzen war; wo alles fortfiel, was einer besessen hatte: Geld – Macht – Ruhm – Glück – wo nur mehr das übrigblieb, was ein Mensch nicht „haben“ kann, sondern was er „sein“ muß: was übrigblieb, war der Mensch selbst – verbrannt vom Schmerz und durchglüht vom Leid, wurde er eingeschmolzen auf das Wesentliche in ihm, auf das Menschliche.

*Was also ist der Mensch?* So fragen wir nochmals. – Er ist ein Wesen, das immer *entscheidet*, was es ist. Ein Wesen, das in sich gleichermaßen die Möglichkeit birgt, auf das Niveau eines Tieres herabzusinken oder sich zu einem heiligmäßigen Leben aufzuschwingen. Der Mensch ist jenes Wesen, das immerhin die Gaskammern erfunden hat; aber er ist zugleich auch jenes Wesen, das in eben diese Gaskammern hineingeschritten ist in aufrechter Haltung und das Vaterunser oder das jüdische Sterbegebet auf den Lippen.

*Das also ist der Mensch.* Und nun wissen wir auch die Antwort auf die Frage, die wir uns im Anfang gestellt: Was ist der Mensch, *daß wir seiner gedenken?* „Er ist ein Schilfrohr“ hat Pascal gesagt, „– aber ein Schilfrohr, das denkt!“ Und dieses Denken, dieses Bewußtsein, dieses Verantwortlichsein –, es macht die Würde des Menschen aus, die Würde jedes einzelnen Menschen. Und es liegt ganz und gar immer nur *am einzelnen* Menschen, ob er sie mit Füßen tritt – oder ob er sie wahrt. So wie das eine das persönliche Verdienst eines Menschen ausmacht – so das andere seine persönliche Schuld. Und *es gibt *nur *persönliche* Schuld! Niemals aber dürfte die Rede sein von Kollektivschuld! Freilich: es gibt auch die persönliche Schuld eines Menschen, der zwar „nichts getan“ hat – aber so manches unterlassen; unterlassen aus Angst um sich selbst oder aus dem Zittern um seine Angehörigen. Wer aber einem solchen Menschen zum Vorwurf machen will, daß er ein „Feigling“ war, der müßte zuvor für seine eigene Person unter Beweis gestellt haben, daß er selber in der gleichen Situation ein Held gewesen.

Doch ist es nicht besser, nicht allzusehr mit andern ins Gericht zu gehen? – Paul Valéry hat einmal gesagt: „Si nous jugeons et accusons, le fonds n’est pas atteint“ – solange wir noch richten und anklagen, ist der Grund nicht erreicht. Und so wollen wir denn nicht nur gedenken, der Toten, sondern auch verzeihen, den Lebenden. So wie wir den Toten die Hand reichen, hinweg über alle Gräber, so wollen wir sie auch den Lebenden entgegenstrecken – hinweg über allen Haß. Und wenn wir sprechen: Ehre sei den Toten – so wollen wir auch hinzusetzen: – und Friede allen Lebenden, *die guten Willens sind*.

#### Gisela Gerbel

The dermatologist, born in 1895, was interned in Theresienstadt at the same time as Frankl and murdered there in 1942.^28^

#### Josef Plautus

Plautus, a doctor for the poor born in 1884, was known as the “Angel of Ottakring”. In 1944, he was sent to the left at Auschwitz train station in front of Frankl’s eyes and was killed in a gas chamber shortly afterwards.^29^

#### Hans Lamberg

The physician, born in 1891, died before Frankl’s eyes in a Dachau camp barracks in 1945.

#### Martha Rappaport

The psychiatrist, born in 1890, initially worked as an assistant doctor at Wagner-Jauregg’s clinic. From 1940, she was Frankl’s colleague at Rothschild Hospital in Vienna. She was deported and murdered in 1942.

#### Paul Fürst

Fürst, a surgeon born in 1910, was also Frankl’s colleague at Rothschild Hospital. He was murdered in Auschwitz in 1944.^30^

## Footnotes

1 Archiv der Universität Wien (Vienna University Archive) (UAW hereafter), Med. Fak. PA Felix Mandl 336, Fol 13 and Fol 20.

2 During the Nazi era Konrad Lorenz was a member of the NSDAP and worked for the Nazi Party Office of Racial Policy [26]. He had no verifiable connection to the College of Physicians during the interwar period and the Nazi era. However, it is worth noting that during the Nazi era, his father Adolf Lorenz (1854–1946) received the Billroth Prize (not identical to the Billroth Medal) at a ceremony held at the Billrothhaus in 1944 on the occasion of his 90th birthday (Fotoarchiv Heinrich Hoffmann, hoff-50553) [27].

3 This article, focusing on the persecution of the Jewish members of the College of Physicians, is the first in a planned series of three.

4 We would like to thank Barbara Sauer and Ilse Reiter-Zatloukal for providing the data they collected as part of the project “Doctors in Austria 1938-1945 – Disenfranchisement, Expulsion, Murder” (https://www.nationalfonds.org/detailansicht/3916), which was of central importance for the statistical analyses. The documentation was based on Adolf Irtl’s official membership list for 1938, which was compared with this data and, in a few cases, with other data as well. In this context, we would also like to refer to other projects that were very valuable to our endeavour: “Memorial Book for the Victims of National Socialism at the University of Vienna 1938” (Herbert Posch, Katharina Kniefacz, Andreas Huber et al.), https://gedenkbuch.univie.ac.at/en; Van Swieten Blog (Walter Mentzel), https://ub.meduniwien.ac.at/blog/; “Lexicon of Austrian Provenance Research” (Pia Schölnberger, Leonhard Weidinger, Sabine Loitfellner et al.), https://www.lexikon-provenienzforschung.org/en.

5 Archiv der Gesellschaft der Ärzte in Wien (Archives of the College of Physicians in Vienna) (ÄGDA hereafter), minutes of meetings 1938–1945, board meeting, Friday, 25 February 1938.

6 AGDÄ, minutes of meetings 1938-1945, To the Berliner Medizinische Gesellschaft [Adolf Irtl]. Vienna, 18 March 1938.

7 Otto Planner-Plann became chairman of the Medical Society of Vienna after the restructuring process was completed. After the *Anschluss* he was Gauamtsleiter of the NSDAP Gauamt für Volksgesundheit and Gauärzteführer. He joined the NSDAP in 1933 and had been a member of the SS since 1934. From September 1937, he was chairman of the National Socialist German Doctors’ League in Vienna. He was authorised to perform surgical infertility treatment on men at the Gersthof Women’s Clinic in Vienna, of which he was the director. In his denazification process, it was not possible to find out whether he made use of this authorisation. Planner-Plan also served as an medical expert and medical assessor (Gutachter und ärztlicher Beisitzer) for the Hereditary Health Court [51, 52]. At the beginning of 1944, he was replaced by Walter Ekhart as Gauamtsleiter für Volksgesundheit [53].

8 AGDÄ, minutes of meetings 1938-1945, minute of meeting, 9 June 1938.

9 AGDÄ, minutes of meetings 1938-1945, new statutes, 21 June 1938.

10 AGDÄ, cash books and accounting books. Folder 1938. Membership fees 1938.

11 The one-year project started on 1 September 2024 and is supported by the City of Vienna’s Department of Cultural Affairs (MA 7), the Cultural Fund of the 9th District, the Future Fund of the Republic of Austria (project P24-5606), the National Fund of the Republic of Austria for Victims of National Socialism and the College of Physicians in Vienna.

12 AGDÄ, Files (Correspondences): 1837–1938, 1938. Carl Fleischmann to Adolf Irtl, Wien, Felix Mottlstrasse 8a, 16.03.1938. Postcard.

13 AGDÄ, Files (Correspondences): 1837–1938, 1938. Letter by Julius Schnitzlers to Anton Eiselsberg, Wien, Laubengasse 18, 15 March 1938.

14 AGDÄ, minutes of meetings 1938–1945, Report on the Library of the College of Physicians in Vienna [1938].

15 AGDÄ, Files: 1837–1938, 1938. Notice by Fischer to the College of Physicians, Vienna, 29 September 1938.

16 Universitätsbibliothek Medizinische Universität Wien, Zweigbibliothek für Geschichte der Medizin (University Library of the Medical University of Vienna, Collection of the Special Library for Medical History) Fischer, Curriculum. Undated.

17 Ibid.

18 Josephinum – Medizinische Universität Wien (Josephinum – Medical University of Vienna) (MUW hereafter), MUW-HS-001984/11; Obituary Isidor Fischer.

19 Wellcome Collection, London, MS.8062, Geographical Names in Medical Terminology (edited by L.T. Morton and H. Pollak), 1 bundle Typescript, 1943, Presented by L.T. Morton, 1975.

20 AGDÄ, minutes of meetings 1938–1945, meeting, 19 October 1945.

21 UAW, Med. Fak. Med. S 17.16.1 – Med. S 17.16.5. Appointment to the Chair of Forensic Medicine. Sch. 118. Leopold Arzt an Fritz Reuter, Wien, 09.05.1945.

22 AGDÄ, minutes of meetings 1938–1945, meeting, 19 October 1945.

23 AGDÄ, minutes of meetings 1938–1945, meeting, 19 October 1945.

24 MUW-AS-003290. Memoirs Wagner-Jauregg. Page 26.

25 MUW-AS-003290. Memoirs Wagner-Jauregg, Page 17.
